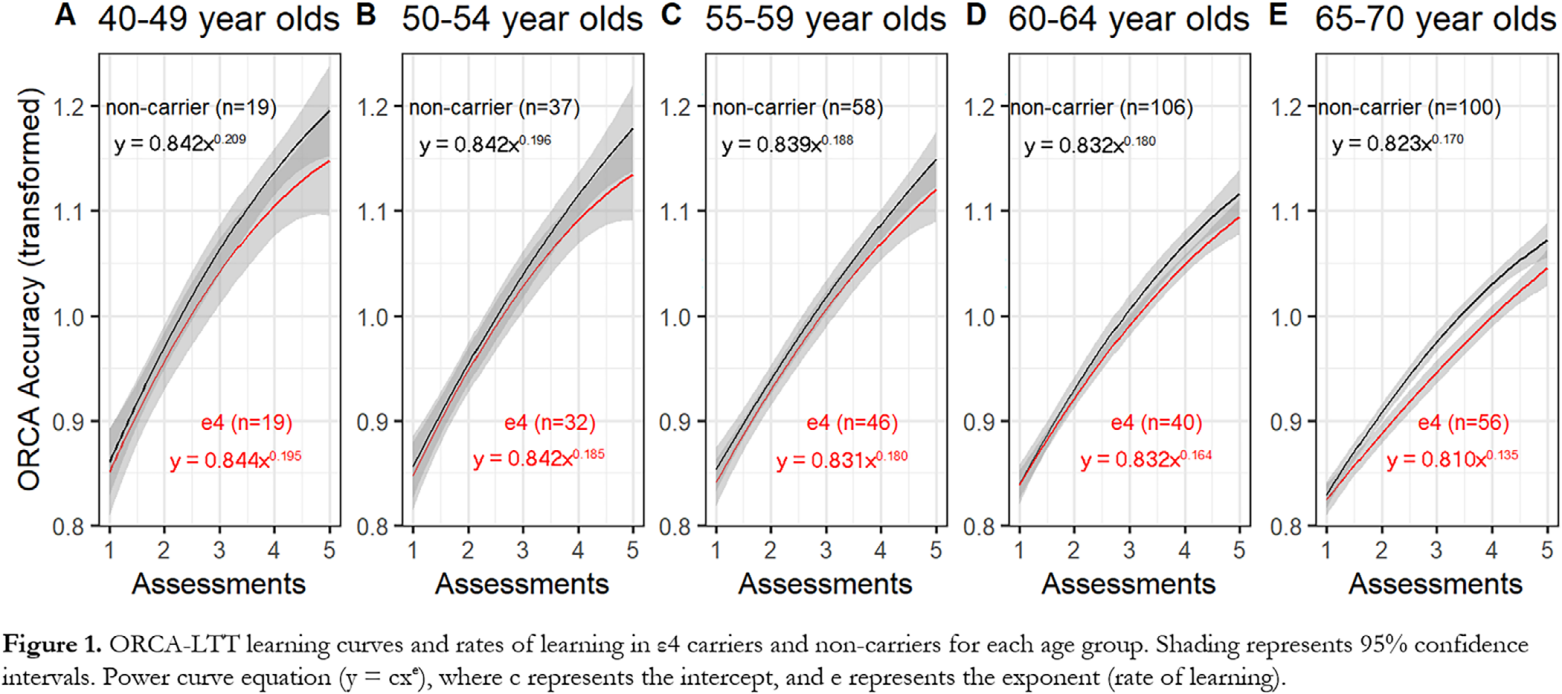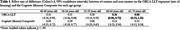# Learning deficits in late middle‐aged *APOE* ε4 carriers: Implications for emergent Alzheimer’s disease

**DOI:** 10.1002/alz.086314

**Published:** 2025-01-03

**Authors:** Yen Ying Lim, Emily Rosenich, Maya Norfolk, Paul Maruff

**Affiliations:** ^1^ Turner Institute for Brain and Mental Health, School of Psychological Sciences, Monash University, Melbourne, VIC Australia; ^2^ Turner Institute for Brain and Mental Health, Monash University, Melbourne, VIC Australia; ^3^ Cogstate Ltd., Melbourne, VIC Australia

## Abstract

**Background:**

Recent studies suggest learning deficits are the earliest cognitive abnormality to emerge in Alzheimer’s disease (AD). Using the Online Repeatable Cognitive Assessment‐Language Learning Test (ORCA‐LLT), cognitively unimpaired (CU) Aβ+ older adults showed a substantial (d∼2) learning deficit compared to Aβ‐ controls. This deficit was six times greater than that observed from reduced practice effects after 9+ years of assessment with conventional neuropsychological memory tests (d∼0.3). A similar but smaller learning deficit (d∼1.2) was also observed in CU Aβ‐ ε4 carriers compared to matched non‐carriers, consistent with observations that Aβ accumulation has begun in this group. We aimed to determine the development of AD‐related learning deficits by examining the effect of age and *APOE* ε4 on the ORCA‐LLT in CU middle‐aged adults.

**Method:**

CU adults (n = 513) provided saliva samples for *APOE* genotyping, and completed the ORCA‐LLT, which required participants to learn associations between 50 Chinese characters and their English language equivalents over 5 days. Learning curves were computed for ORCA‐LLT performance accuracy across days, with learning rate defined using the exponent estimated from each power curve. The Cogstate Brief Battery was also completed and provided the Cogstate Learning/Working‐Memory Composite. All assessments were completed remotely and unsupervised on an online platform.

**Result:**

Learning curves were evident for all groups, though learning rates slowed with increasing age (Fig 1). Learning rates were slower in ε4 carriers compared to non‐carriers in the 65‐70 (d∼0.9) and 60‐64‐year age groups (d∼0.4). Learning rates were equivalent between ε4 carriers and non‐carriers in all other age groups (Fig 1, Table 1). No differences were observed between ε4 carriers and non‐carriers on the Cogstate Learning/Working‐Memory Composite for any age group (Table 1).

**Conclusion:**

In late middle‐aged adults, ε4 was associated with a large reduction in ability to learn novel information. This impairment was not evident in performance on conventional cognitive tests. It is likely that Aβ levels in this group are increasing abnormally or have reached threshold for abnormality. Placed in the context of our previous observations, these data support the hypothesis that the earliest cognitive abnormality to reflect AD pathophysiological processes is a reduced ability to benefit from new information.